# Metal 3D Printing Is Showing Its Mettle

**DOI:** 10.1021/acscentsci.2c01030

**Published:** 2022-09-08

**Authors:** Prachi Patel

As a US Marine Corps combat engineer serving in Iraq, Jim Monroe used MacGyver-like hacks to repair mine rollers. Meant to detonate and clear mines, these heavy studded wheels are mounted on the front of tanks.

“They were modular devices held together by small pins, but we didn’t have extra pins so we’d use zip ties to hold the parts together,” says Monroe, now senior director of additive manufacturing at Spee3D.

The start-up, whose name is pronounced “speed 3D,” develops 3D-printing technology for metal. “If we’d had a metal 3D printer, we could just print those pins and repair our mine rollers,” Monroe says.

That sentiment is most likely shared by many soldiers and surgeons—or anyone who’s assembling Ikea furniture and looking for a missing screw. Users can employ 3D printing to create complex parts on demand without metallurgy or machining expertise. For soldiers and medics, this could mean creating a lifesaving solution in the field. For manufacturers, the ability to quickly print prototypes and replacement parts for testing could save thousands of dollars and months of R&D.

Three-dimensional printers are now found in retail stores, on shop floors, and in schools. But materials for 3D printing have largely come from the realm of more moldable—and generally weaker—polymers since the 1980s, when such printing began.

Printing metal is trickier. Common printing techniques yield porous materials that lack the ordered crystalline microstructure of bulk metal and thus are unsuitable for applications like engines and vehicle bodies. Printing metal is also expensive, slow, and energy intensive. Most important, printed objects can’t match the strength, density, and durability of traditionally produced metal.

Be it large boat hulls, airplane wing flaps, or intricate structures that go into cars or robots, start-ups like Spee3D want to give customers a way to quickly and cheaply create high-performance components for prototyping and real-world use. And as these firms expand the universe of facilities capable of shaping metal, questions arise about how to ensure safety.

For centuries, humans have primarily employed two techniques to shape metal: casting and forging. Casting involves melting metal to pour it into a mold and is generally used for quick production. Notably, as the metal melts and resolidifies, it can absorb oxygen and become brittle.

Forging, which shapes hot-but-still-solid metal by hammering or pressing, is used to make high-performance parts. That’s because deformation of the solid material results in smaller grains—the microscopic crystals that make up a bulk metal—which translate to stronger and more dense materials.

Powder bed fusion, which emerged in the 1990s, is now the most widely used technique for printing metal parts. The technique relies on a programmable laser or electron beam to fuse metal powder in an inert-gas environment to create a solid object.

At a microscopic level, fusion is much like traditional casting. In both processes, the metal is melted and then quickly solidifies; that leaves behind a microstructure full of pores and cracks. Those flaws make powder bed fusion less than ideal for printing high-performance materials—at least without a follow-up, energy-intensive heat treatment to strengthen them. Airplane parts, for example, need to have very low levels of defects, says Hang Yu, a materials scientist and engineer at Virginia Tech. “You can make complex geometries with powder bed fusion,” he says. “But it has been used mainly for research and demonstration purposes.”

For performance parts, Spee3D has advanced a technique called cold spray deposition, which uses a pressurized jet of air to shoot metal powder at supersonic speeds onto a surface. The particles in the powder, each tens of micrometers wide, slam into the surface and fuse with one another on impact. Cold spray deposition has been used for decades to coat things with metal.

To 3D print complex parts, Spee3D adapted the technique to combine a nozzle that shoots metal powder with a robotic arm that moves and tilts a target plate in front of the powder stream to create custom shapes, Monroe explains. Because Spee3D’s technique is performed in open air with no inert gas involved, the machines can print very large parts.

As its name implies, Spee3D aims to give customers the advantage of printing metal parts much more quickly than is possible with powder bed fusion. Spee3D’s solid-state process avoids the melting and rapid cooling that results in cracks and defects. So, depending on the application, parts are ready to use straight off the printer—perhaps not as strong as if they were forged, but better than if they were cast. “Our customers tell us they were printing copper magnets in 3 days, and they can now do it in 1.5 hours,” Monroe says.

At the end of August, the US Navy tested Spee3D machines at sea to print ship components. “The military needs replacement parts,” Monroe says. “Imagine being able to print an object in minutes. At the end, you get a part that’s stronger than cast part.”

**Figure:**
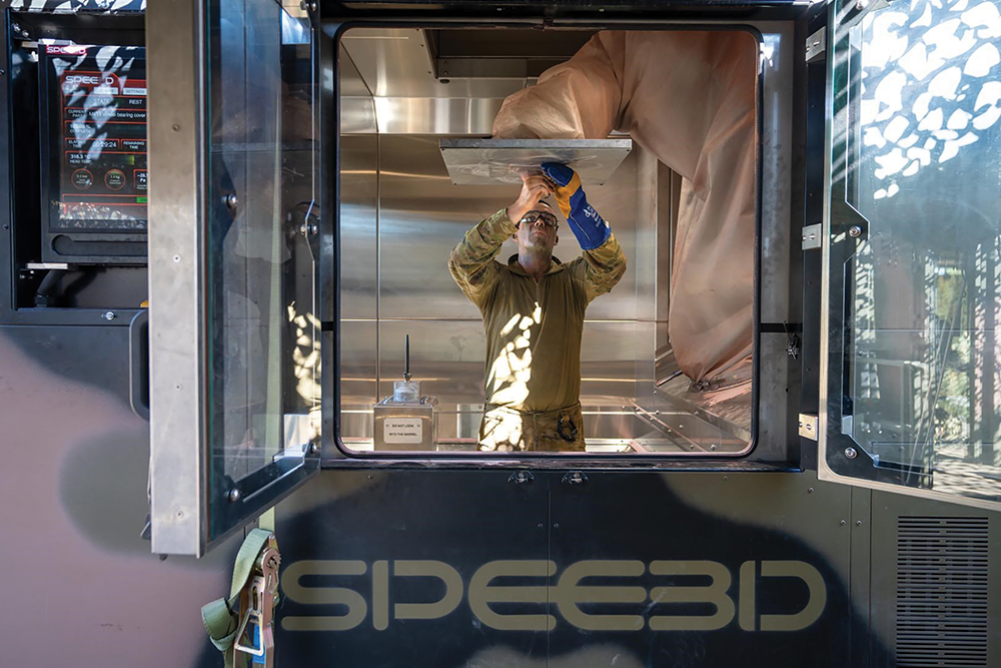
An Australian Army soldier removes a newly 3D-printed metal part from Spee3D’s printer during a trial of the technology in a field environment. Credit: Spee3D

**Figure:**
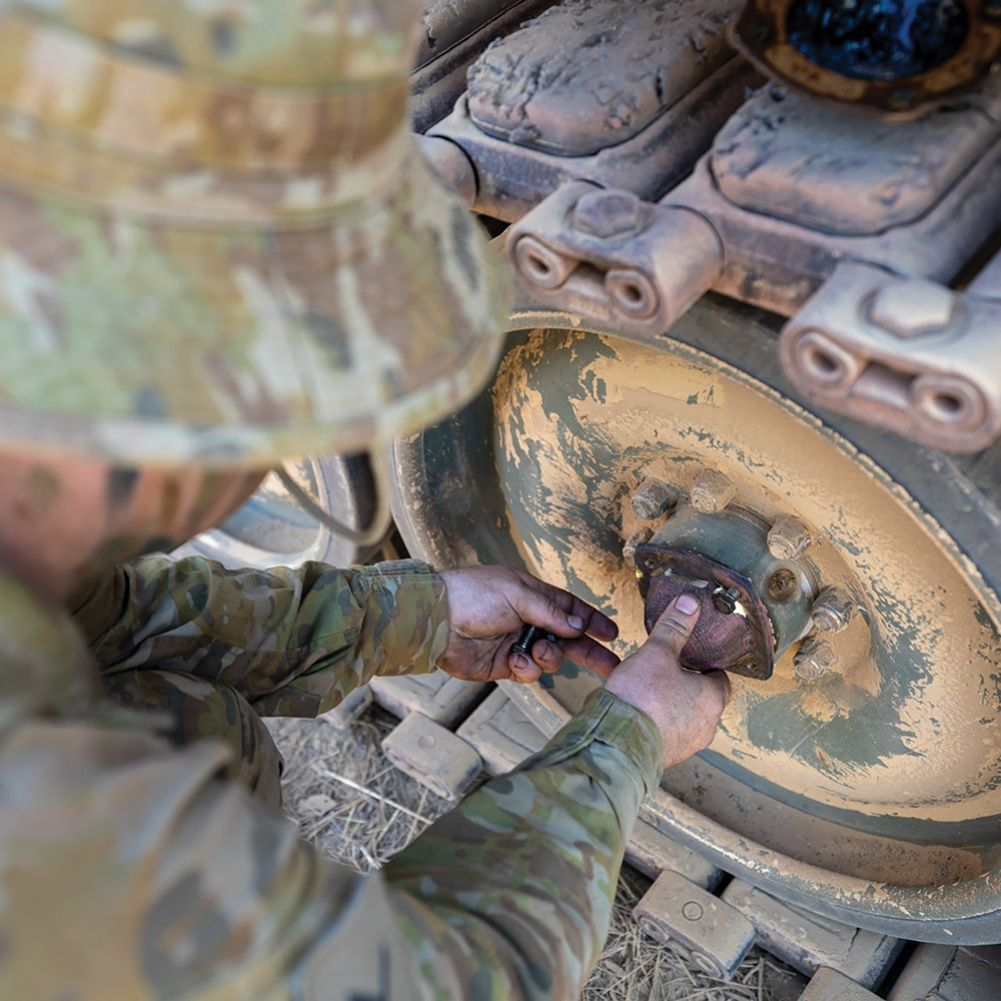
In a 12-month trial of Spee3D’s metal 3D-printing technology, Australian Army soldiers fabricated parts like armored vehicle wheel-bearing covers and tested them in the field. Credit: Spee3D

While Spee3D pushes time limits, Meld Manufacturing is working to print parts with the strength and density of forged metal.

CEO Nanci Hardwick, who founded Meld in 2018, describes its process as an extreme version of bending a paper clip back and forth. Meld’s machines start with metal bars and use force and a stirring action to heat and deform the material instead of melting it. The result of such friction-stir deposition is thin layers of metal pressed together.

**Figure:**
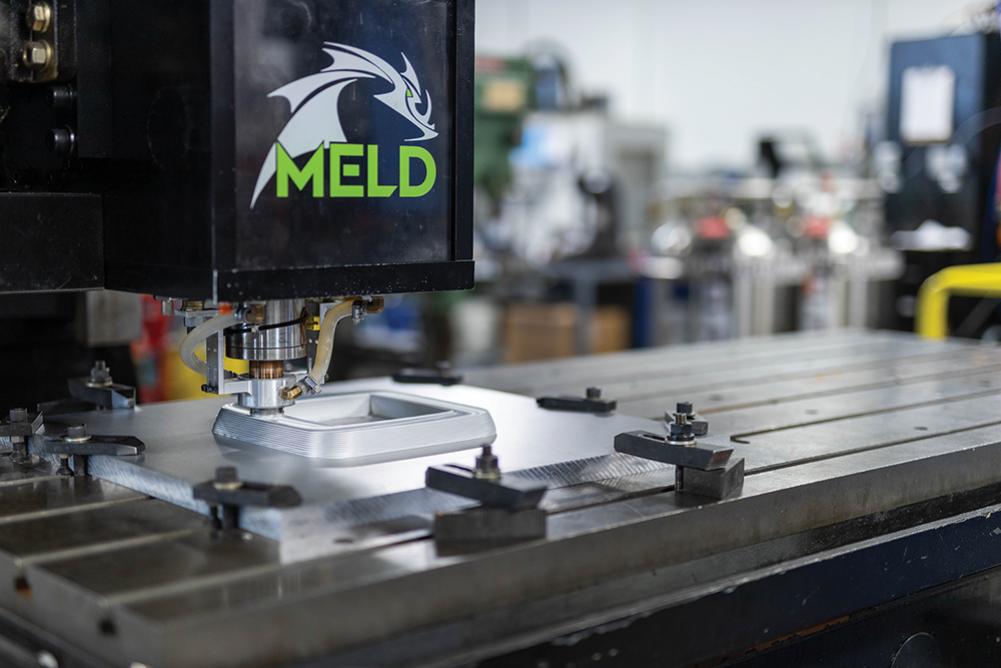
Meld Manufacturing’s friction-stir deposition process bonds metal layers together into strong, dense parts using heat and pressure; the open-air technology can print very large objects. Credit: Meld Manufacturing

Under the sheer force and heat, the surfaces chemically bond and form a dense, monolithic structure. The stirring also results in finer grains that are one to two orders of magnitude smaller than those in the metal fed into the machine, resulting in higher strength, corrosion resistance, and wear resistance. “We’re capable of printing at unsurpassed speed, at any size, with forged properties,” Hardwick says. “I liken it to printing wrought iron.”

The metal bars fed into the printer are more economical and safer than metal powders, which come with fire risks and can be inhaled by operators. Bars are also more familiar to manufacturers, Hardwick says.

Through a research partnership with Yu at Virginia Tech, Meld hopes to gain an in-depth understanding of the friction-stir deposition process. Yu’s team uses electron microscopy, electron diffraction, and other techniques, bolstered by computer modeling, to examine how material flows and distorts under heat and how its microstructure evolves.

In addition to being able to print large parts, Meld’s technology can be used for repairs because of how well the printed metal bonds with existing metal layers. That would allow technicians to fix corrosion damage on high-strength aluminum airplane frames or soldiers to fill holes in a damaged truck or boat.

The various applications of friction-stir deposition have captured the attention of the aerospace, defense, and shipbuilding industries. In June, Meld won a US Army contract to build a metal printer large enough to print military vehicle hulls. Visitors to the company and to Yu’s lab include the helicopter maker Sikorsky and the private aerospace leader Blue Origin. “Customers are asking to keep pushing our equipment to print bigger and bigger parts,” Hardwick says.

Both Spee3D and Meld’s technologies can print several kilograms per hour of stainless steel and aluminum—tens of times faster than powder bed fusion can. And because they work at lower temperatures than fusion systems, they reduce the energy footprint of 3D-printed metal.

Nevertheless, cold spray deposition and friction-stir deposition are not good for printing complicated structures like parts containing fine channels, trusses, or mesh. And while the processes can print many different metals and alloys, they can currently handle only relatively soft ones, such as aluminum, copper, titanium, and stainless steel. Harder materials like high-strength steel present a challenge.

Yu says the fundamental knowledge his group is gaining from process and microstructure analysis should help expand Meld’s repertoire. “We want to understand the process in depth and address the difficult problems,” he says.

Working through those problems and balancing strength, print speed, and size is vital to produce heavy-duty equipment, but that isn’t the only application. For many manufacturers, metal 3D printing is a way to turn vision into reality by making metal prototypes as cheaply and easily as plastic parts.

Boston-based Markforged has sold affordable desktop 3D printers for plastics and composites since 2013. It adapted its filament extrusion technique for metal printing in 2018, focusing on low cost and ease of use. The printer nozzle extrudes a heated filament composed of metal powder, polymer, and wax to build parts a layer at a time.

**Figure:**
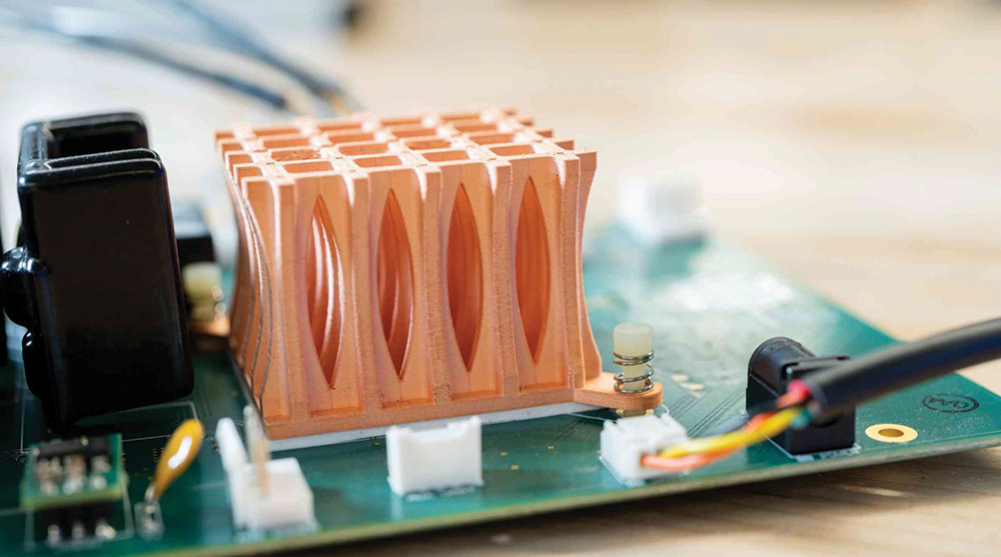
Being able to 3D print with pure copper allows manufacturers to quickly and easily make intricate structures, like this heatsink used to dissipate heat in computers. Credit: Markforged

After a part is printed, a quick solvent wash removes the wax, and a day of sintering in a furnace burns off the polymer to give the finished part, according to Ben Gallup, R&D engineering manager at Markforged. “It’s easy for companies to adopt the technology and build on their skill level of plastic 3D printing,” Gallup says.

In July, Markforged bought competitor Digital Metal, which makes printers based on a similar technology called binder jetting. These printers work much like inkjet printers, laying down alternate layers of metal powder and binder to build a part. “The resolution of binder jet parts is fantastically sharp. It’s just astounding,” Gallup says. The printers can also produce parts 100 times as fast as powder bed fusion systems.

And Markforged’s machines cost $150,000, compared with many hundreds of thousands of dollars for powder bed fusion systems. “It’s a game changer for metal 3D printing because it really brought down cost,” says Ross Adams, the company’s metal product manager. “It helps people get their toes in the water,” he adds. “More companies are starting to understand the value of metal additive manufacturing. You just need a digital file and a printer that can churn out parts for you.”

The new ease of production has a potential downside in the form of illicit use. Ever-cheaper plastic 3D printers and downloadable gun blueprints have created a new route to homemade guns and have led to legal battles to stop their proliferation. Plastic guns have been shown to shatter after firing a few shots, but a 3D-printed metal gun could be more robust. In 2013, the start-up Solid Concepts used a type of powder bed fusion to make the first 3D-printed metal shotgun to showcase the technology’s capability. For now, however, it’s much easier and less costly to assemble an untraceable metal gun out of readily available parts than to 3D print one, according to Philip Cook, a gun policy expert at Duke University.

Metal 3D-printing companies say they will continue to focus on space exploration and military endeavors. For the military, Monroe says, the value in being able to print spare parts and specialty components in remote locations and at sea cannot be overstated. Soldiers given Spee3D printers have come up with cutting-edge applications, he adds. For example, he says, “they’re mixing powders to get nonconductive alloys for fuel nozzles so they can fill tanks without a risk of sparks.”

Now, with funding from the Australian government, Monroe says Spee3D plans to build low-cost rocket engines, which could help that nation’s budding space industry take off. “People didn’t think it could be done with cold spray, but we’ve proven it.”

*Prachi Patel is a freelance contributor to**Chemical & Engineering News**, the weekly newsmagazine of the American Chemical Society.*

